# Dietary fibers to boost endogenous GLP-1 secretion and satiety: a scoping review

**DOI:** 10.3389/fendo.2026.1880500

**Published:** 2026-07-15

**Authors:** Jelle C. B. C. de Jong, Milou G. W. Lentjes, Karleen F. Pietersma, Wilrike J. Pasman, Suzan Wopereis, Femke P. M. Hoevenaars

**Affiliations:** 1 Department of Microbiology and Systems Biology, The Netherlands Organization for Applied Scientific Research (TNO), Leiden, Netherlands; 2 Department of Biomedical Signals and Systems, University of Twente, Enschede, Netherlands

**Keywords:** appetite, functional foods, incretins, nutrition, prebiotics, supplements, weight regain

## Abstract

**Background:**

Dietary fibers can stimulate endogenous glucagon−like peptide−1 (GLP−1) secretion through microbial fermentation and gut hormone signaling, potentially enhancing satiety and supporting weight management. Given the growing interest in non−pharmacological strategies to complement or support tapering of GLP−1 receptor agonist therapy, a structured overview of the human evidence is needed.

**Methods:**

A pre−registered scoping review was conducted using PubMed, Scopus and Cochrane Central. Randomized controlled trials in adults assessing circulating GLP−1 concentrations and satiety following supplementation with a single, well−defined dietary fiber were included. Fiber types were categorized based on structural characteristics. Outcomes were summarized qualitatively across fiber categories.

**Results:**

In total, 1049 papers were screened and 49 publications comprising 52 studies (total n=1,085 participants; median sample size per study=19) were included. Most studies were acute interventions (71%) and conducted in Western populations. Studies reporting increased GLP-1 showed a non-significant tendency to also report increased satiety (OR = 2.95, 95% CI: 0.87–9.98). Dextrins stood out as one of the few fiber categories showing robust effects on both GLP−1 (4 positive studies) and satiety (5 positive studies). Other fibers, such as β−glucans and mannans, showed more uniform effects on satiety or GLP−1, respectively, but did not consistently affect both outcomes simultaneously.

**Conclusions:**

Although these findings identify dextrins as a promising dietary fiber candidate for future research, the evidence remains constrained by small sample sizes, short interventions, and substantial heterogeneity. Longer−term studies in free−living conditions, including periods of GLP-1 receptor agonist tapering, are needed to capture microbiota adaptation and generate robust real−world evidence.

**Systematic review registration:**

https://osf.io/cnw4e/overview.

## 1 Introduction

GLP-1 receptor agonists (GLP-1RAs) can induce 10–20% weight loss when administered once weekly, primarily by reducing appetite and prolonging satiety (1, 2). Despite their effectiveness, long-term use of GLP-1RAs is often limited. Individuals may discontinue treatment for a variety of reasons, including side-effects, loss of insurance coverage, cost considerations or other difficulties with long-term adherence. Following discontinuation, people treated with GLP-1RAs are expected to gradually return to baseline body weight within approximately 1.7 years (3, 4). These challenges highlight the need for alternative strategies that support the tapering of GLP-1RA therapy while maintaining achieved weight loss and associated health benefits. Lifestyle-based approaches are particularly relevant in this context, as they can be sustained over longer periods and integrated into routine obesity or metabolic disease care. Dietary strategies in particular, offer a low-risk and broadly applicable means to support long-term weight management and metabolic health, both as adjuncts to pharmacotherapy and during medication tapering (4).

Endogenous GLP-1 is released by intestinal L-cells in response to nutrient sensing, bile acid signaling, and microbial metabolites (5). Among these metabolites are short-chain fatty acids (SCFAs), including acetate, propionate, and butyrate, which are produced through the fermentation of dietary fibers in the colon (5). SCFAs activate the free fatty acid receptors FFAR2 (GPR43) and FFAR3 (GPR41) on the intestinal L-cells, thereby stimulating endogenous GLP-1 secretion, with propionate acting as the most potent agonist of these receptors (6, 7). Dietary fibers comprise chemically and physiochemically diverse compounds that differ in monomer composition, glycosidic linkages (α−1,4; α−1,6; β−linkages), solubility, viscosity, and fermentability. These characteristics determine whether fibers primarily influence gastric mechanics, intestinal transit, or colonic fermentation and, consequently, their potential effects on satiety and endogenous GLP−1 secretion. Accordingly, this review applies a previously established 9−category fiber classification (8; **Supplementary Table 1**) to evaluate how structural fiber properties relate to endogenous GLP−1 responses and subjective satiety.

While numerous studies have investigated individual fiber types, findings are heterogeneous and a systematic overview identifying which dietary fibers most effectively enhance endogenous GLP-1 secretion and satiety is currently lacking. As a result, it remains unclear which dietary fibers most consistently enhance endogenous GLP-1 secretion and satiety in clinically relevant populations, limiting translation to dietary recommendations and intervention design. This review therefore summarizes the current evidence on the effect of dietary fiber supplementation or enrichment of food products on endogenous GLP-1 secretion and satiety. We focused on randomized controlled human trials conducted in relevant adult populations, including individuals with overweight, obesity, type 2 diabetes mellitus, or metabolic syndrome, as well as healthy adults as a reference population. Finally, we discussed how these findings may guide the design of larger clinical studies and contribute to the development of personalized care pathways for obesity and metabolic disease, including strategies to support GLP-1RA tapering and the prevention of weight regain.

## 2 Materials and methods

### 2.1 Literature search strategy and study selection

This review, including its applied methodologies, was pre-registered on the Open Science Framework (https://osf.io/cnw4e/overview) on 16 December 2025. An electronic literature search of PubMed, Scopus and Cochrane Central was conducted on the same date using the following search terms: (Dietary Fiber[Mesh] OR Prebiotics[Mesh] OR Polysaccharides[Mesh] OR Inulin[Mesh] OR beta-Glucans[Mesh] OR Psyllium[Mesh] OR Pectins[Mesh] OR “guar gum”[Supplementary Concept] OR “Gum Arabic”[Mesh] OR Cellulose[Mesh] OR Alginates[Mesh] OR “(1-6)-alpha-glucomannan” [Supplementary Concept] OR xanthan gum[Supplementary Concept] OR fiber[tiab] OR fibre[tiab] OR “dietary fiber”[tiab] OR “dietary fibre”[tiab] OR “soluble fiber”[tiab] OR “insoluble fiber”[tiab] OR prebiotic*[tiab] OR inulin[tiab] OR oligofructose[tiab] OR fructooligosaccharide*[tiab] OR galactooligosaccharide*[tiab] OR xylooligosaccharides[tiab] OR fos[tiab] OR gos[tiab] OR xos[tiab] OR isomaltooligosaccharide*[tiab] OR isomaltose[tiab] OR “resistant starch”[tiab] OR “RS2”[tiab] OR “RS3”[tiab] OR “RS4”[tiab] OR “beta-glucan”[tiab] OR psyllium[tiab] OR pectin[tiab] OR “guar gum”[tiab] OR “partially hydrolyzed guar gum”[tiab] OR phgg[tiab] OR fibersol[tiab] OR “arabic gum”[tiab] OR “acacia gum”[tiab] OR “gum arabic”[tiab] OR cellulose[tiab] OR hemicellulose[tiab] OR arabinoxylan*[tiab] OR polydextrose[tiab] OR dextrin[tiab] OR “wheat dextrin”[tiab] OR glucomannan[tiab] OR konjac[tiab] OR alginate[tiab] OR alginic[tiab] OR “sodium alginate”[tiab] OR “xanthan gum”[tiab] OR “locust bean gum”[tiab] OR “carob gum”[tiab] OR “pectic oligosaccharide*”[tiab]) AND (Glucagon-Like Peptide 1[Mesh] OR Incretins[Mesh] OR glp1[tiab] OR “glp-1”[tiab] OR “glucagon-like peptide-1”[tiab] OR incretin*[tiab]) AND (Satiety Response[Mesh] OR Appetite Regulation[Mesh] OR Food Intake[Mesh] OR Feeding Behavior[Mesh] OR satiety[tiab] OR appetite[tiab] OR fullness[tiab] OR hunger[tiab] OR “energy intake”[tiab] OR “food intake”[tiab] OR “ad libitum”[tiab]) AND (Placebos[Mesh] OR maltodextrin[tiab] OR cellulose[tiab] OR microcrystalline cellulose[tiab] OR starch[tiab] OR “corn starch”[tiab] OR “potato starch”[tiab] OR dextrose[tiab] OR sucrose[tiab] OR fructose[tiab] OR glucose[tiab] OR polydextrose[tiab] OR carboxymethylcellulose[tiab]).

There was no cut-off criterion for publication date. Conference abstracts, trial protocols, non-English articles, and preprints were excluded. Records were initially screened based on title and abstract against the predefined PICO criteria and subsequently assessed at the full-text level.

PICO criteria were defined as:

- Population: Healthy adult subjects or adult subjects with overweight, obesity, diabetes type II or metabolic syndrome.
- Intervention: Supplementation with a specific fiber or enrichment of food products with a specific fiber. We exclude studies using food products enriched with mixed fibers.
- Control: Control interventions can be isocaloric food products without enrichment of the tested fiber or with a control fiber (e.g., maltodextrin). Both separate control groups as well as (single-arm) cross-over designs are allowed.
- Outcome: Blood GLP-1 concentrations AND satiety as measured through a Visual Analogue Scale or a Likert Scale.

### 2.2 Data extraction

Extracted data included first author, year of publication, country in which the study was conducted, study design, population characteristics, sample size, sex distribution, age, body mass index (BMI), type of dietary fiber tested, dietary fiber dose and intervention duration, control condition, and the effects of the dietary fiber on circulating GLP-1 concentrations and subjective satiety. Subjective satiety was assessed across studies using visual analogue scales or Likert scales capturing multiple appetite-related sensations, including hunger, desire to eat, fullness, satiation, prospective food consumption, and satisfaction. A dietary fiber was considered to have a significant effect on subjective satiety if at least one of these measures showed a statistically significant favorable effect. Due to substantial heterogeneity in outcome reporting across studies, including differences in GLP-1 assays, satiety measurement scales, and reporting formats, outcomes were harmonized into directional categories (increase vs no increase/decrease). This approach enabled consistent comparison across studies within the scope of this scoping review.

Data extraction was performed independently by two reviewers. In cases of disagreement that could not be resolved through discussion, a third independent reviewer made the final decision regarding data extraction. Some articles reported data from multiple interventions (e.g., testing multiple dietary fiber types), in such cases each intervention was treated as an independent study, even though they originated from the same publication. As this study was designed as a scoping review, a formal risk-of-bias or certainty-of-evidence assessment was not performed, in line with the aim to map the available evidence rather than to quantitatively synthesise study quality.

### 2.3 Fiber categorization

Fiber types were categorized according to the structural classification system described by Wanders et al. (8), in which dietary fibers are categorized primarily based on their chemical structure. Within this framework, fibers consisting of glycose polymers with predominantly α−1,4 linkages were classified as resistant starch. Fibers containing other α−1,4 linkages, typically generated through the partial hydrolysis of starch, were categorized as dextrins, including resistant dextrins such as polydextrose, which may differ structurally (e.g., by containing α−1,6 linkages) but are grouped together based on similar functional and physicochemical properties, consistent with the framework of Wanders et al. whereas fibers containing other α−1,4 linkages, typically generated through the partial hydrolysis of starch, were categorized as dextrins. Glucose-based polymers characterized by β−linkages were classified as β−glucans. Fibers composed of mainly mannose residues, such as galactomannans and glucomannans, were classified as mannans. Similarly, polymers primarily consisting of fructose units, including inulin, oligofructose and fructooligosaccharides were categorized as fructans. Dietary fiber types dominated by xylose residues, such as arabinoxylan-oligosaccharides or xylan-rich wheat-bran extracts, were assigned to the xylan category. Pectin-rich fibers were grouped as pectins. In addition to these structurally defined groups, cereal-derived arabinoxylan-containing fibers (e.g., rye fiber, corn arabinoxylans, and psyllium) were classified as arabinoxylan-rich. Finally, fibers of marine origin, such as alginate and other seaweed-derived polysaccharides, were grouped as marine polysaccharides.

Each intervention was categorized according to the primary fiber constituent tested. When a publication reported multiple interventions involving different fiber types, each intervention arm was treated as an independent study for the purpose of categorization and analysis.

### 2.4 Statistical analysis

Statistical Statistical analyses were performed using R Statistical Software (version 4.6.0; R Foundation for Statistical Computing, Vienna, Austria, https://www.r-project.org/). To assess whether GLP-1 responses were associated with satiety responses at the study level, we fitted a logistic regression model with satiety response as the binary outcome and GLP-1 response as the binary predictor. Satiety was classified as either increased or not increased, whereas GLP-1 was classified as increased versus no increase or decrease. Model fit was evaluated by comparing the GLP-1 model against an intercept-only model using a likelihood-ratio test. Odds ratios and 95% confidence intervals were estimated from the fitted logistic regression model. As a sensitivity analysis, we repeated the model with fiber category included as a fixed effect to account for heterogeneity between fiber types. The adjusted model was compared with a model including fiber category alone.

## 3 Results

### 3.1 General results

**Figure 1:**
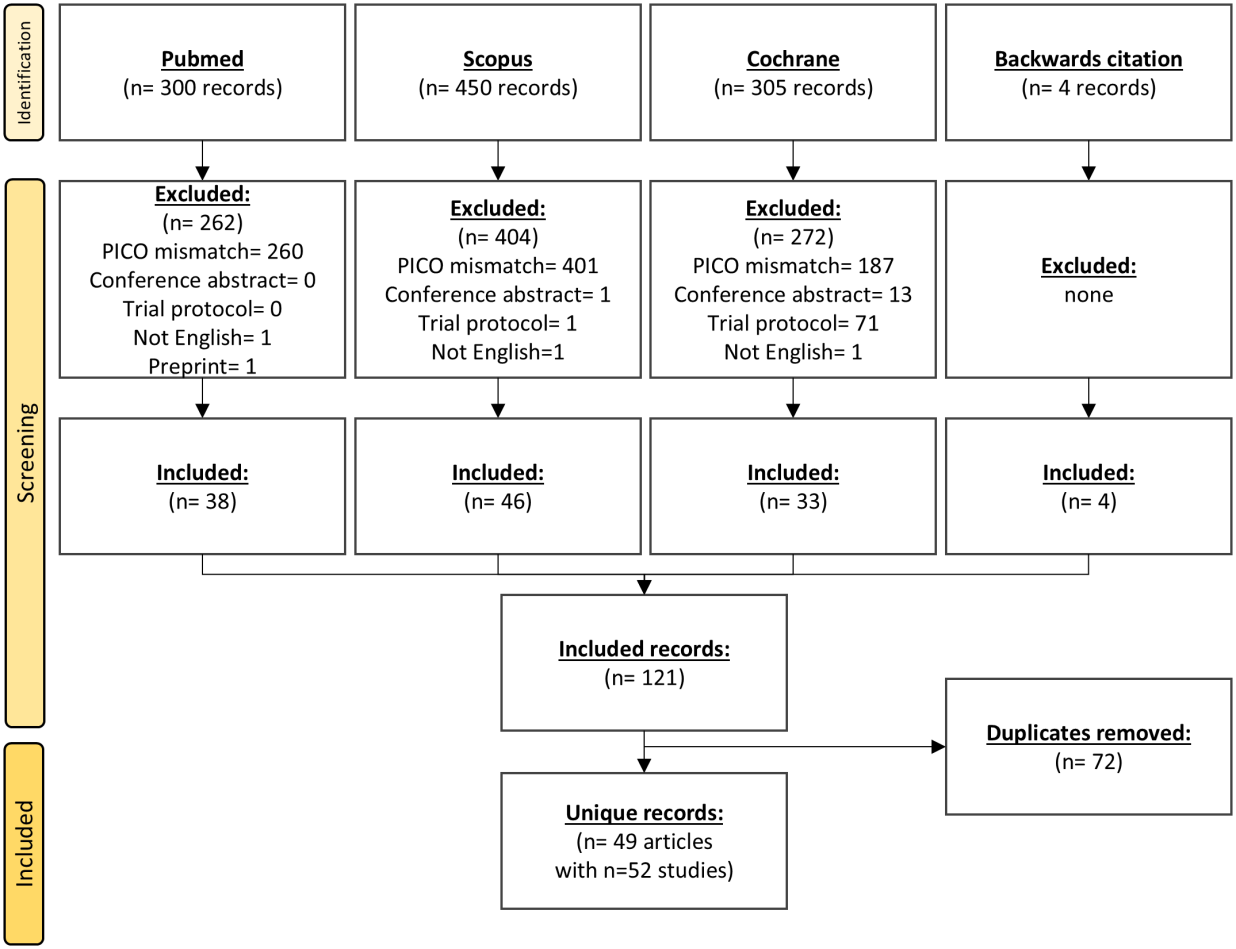
PRISMA flow diagram illustrating the identification, screening, and inclusion of studies. Records were identified through PubMed (n = 300), Scopus (n = 450), Cochrane Central (n = 305), and backward citation searching (n = 4). After exclusion based on predefined criteria, including PICO mismatch, conference abstracts, trial protocols, non-English articles, and preprints, a total of 121 records were retained. Following removal of duplicates (n = 72), 49 unique articles comprising 52 individual studies were included in the final qualitative synthesis.

A total of 300 records were identified through PubMed, 450 through Scopus, and 305 through Cochrane, of which respectively 38, 46, and 33 records met the PICO criteria (**Figure 1**). Four additional publications were identified through a backward search of the references cited in articles reviewed for inclusion. After removing duplicates, a total of 49 unique publications were included. Some publications reported results from multiple interventions that were treated as separate studies; therefore a total of 52 different studies were identified. The details of every included study are provided in **Supplementary Table 2**.

**Figure 2:**
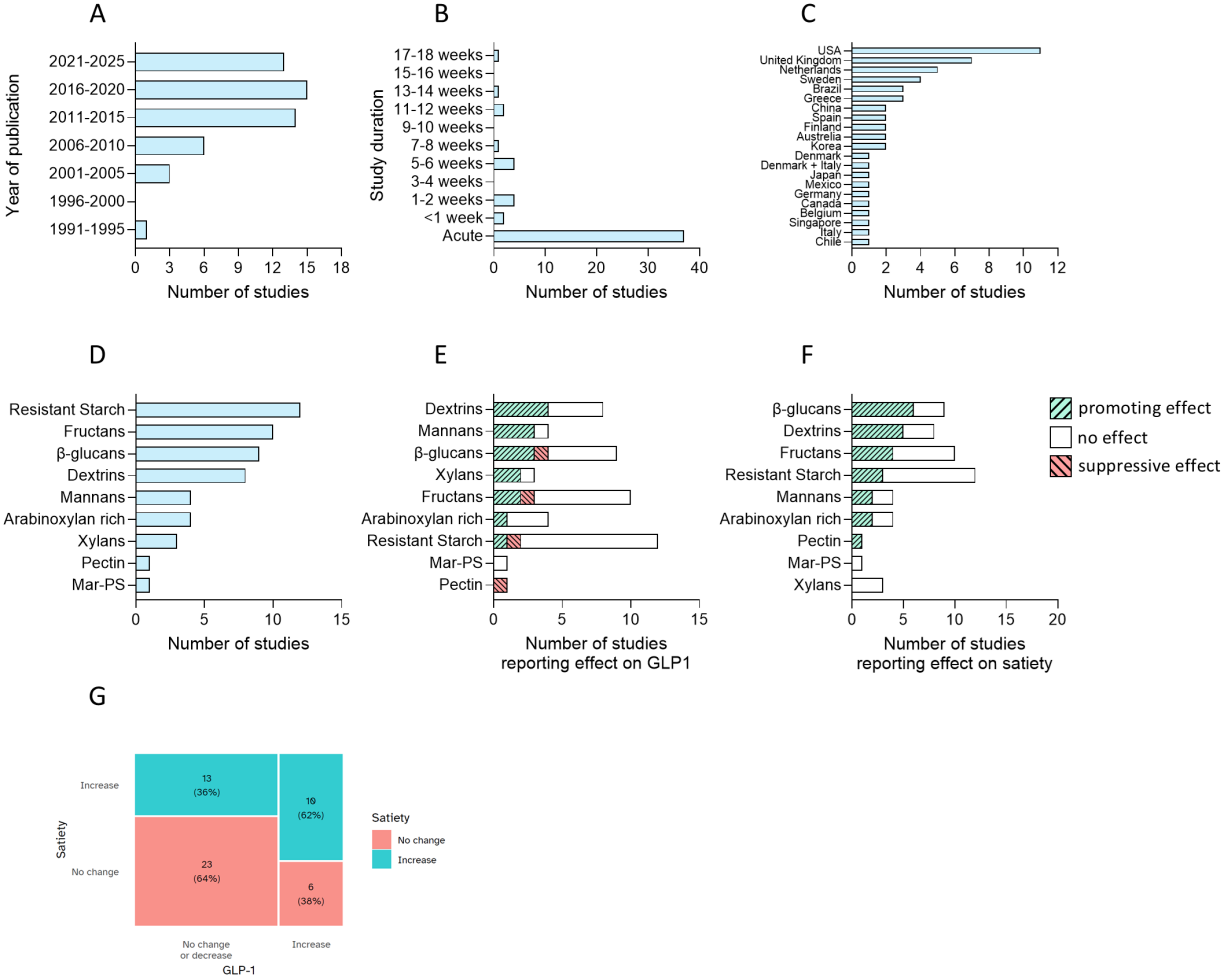
Overview of included studies investigating the effects of dietary fibers on endogenous GLP-1 and satiety outcomes. **(A)** Number of published studies per 5-year period. **(B)** Distribution of study durations, including acute and short- to longer-term interventions. **(C)** Geographic distribution of studies by country. **(D)** Number of studies investigating each fiber category. **(E)** Number of studies reporting effects for each fiber category on GLP-1 concentrations, categorized as promoting (green), no effect (white), or suppressive (red). **(F)** Number of studies reporting effects for each fiber category on satiety, categorized as promoting (green), no effect (white), or suppressive (red). **(G)** Mosaic plot of GLP-1 response (no change/decrease vs. increase) and satiety response (no change vs. increase), showing study counts and percentages.

Across the 52 studies, the combined sample size was 1,085 participants (median per study = 19; IQR = 13; range = 7–58). The majority of included studies were published after 2010, with a clear increase in publication frequency over time, with the highest number of studies published between 2016 and 2020 (**Figure 2A**). Most interventions were of short duration, with a large proportion (71%) of studies assessing acute effects of dietary fiber intake (**Figure 2B**). Among longer-term interventions, study durations most frequently ranged from one to four weeks, while relatively few studies extended beyond eight weeks. Geographically (**Figure 2C**), The United States contributed the largest number of studies (n=12), followed by The United Kingdom (n=7) and The Netherlands (n=5). Several other countries (e.g., Sweden, Brazil and Greece) were represented by smaller numbers of studies.

A wide range of dietary fiber types was investigated (**Figure 2D**). Resistant starch was the most frequently investigated fiber type (n=12), followed by fructans (n=10) and dextrins (n=8). β-glucans, mannans, arabinoxylan-rich fibers, and xylans were moderately represented, whereas pectin, and marine polysaccharides (Mar-PS) were evaluated less frequently. When stratified by outcome, dextrins (4 out of 8 studies) and mannans (3 out of 4 studies) were most frequently associated with reported increases in circulating GLP-1 concentrations (**Figure 2E**). β-glucans (3 out of 9 studies) were reported to increase GLP-1 concentrations, but also reported to suppress GLP-1 concentrations in one study. Fructans (2 out of 10 studies) and xylans (2 out of 3 studies) were reported to increase GLP-1 concentrations in two studies each, but fructans were also reported to suppress GLP-1 concentrations in one study. Arabinoxylan rich and resistant starch fiber types were reported to increase GLP-1 concentrations in only one study, and resistant starch fiber was also reported to suppress GLP-1 concentrations in one study. For mar-PS no effects were reported, and pectin was reported to decrease GLP-1 concentrations in one study.

For satiety outcomes, β-glucans (6 out of 9 studies), dextrins (5 out of 8 studies) and fructans (4 out of 10 studies) were most frequently reported to promote subjective satiety (**Figure 2F**). Resistant starch was the most investigated fiber, with a relatively low number of studies reporting satiety promoting effects (3 out of 12 studies). Other fibers were reported to promote satiety in one or two studies, whereas Mar-PS and xylans did not promote satiety in any of the included studies. Studies reporting increased GLP-1 tended to have higher odds of also reporting increased satiety compared with studies reporting no GLP-1 increase or a decrease, but this association did not reach conventional statistical significance (**Figure 2G**). The odds of a positive satiety response were estimated to be 2.95-fold higher in GLP-1-positive studies, although the confidence interval was wide and included the null value (OR = 2.95, 95% CI: 0.87–9.98). Inclusion of GLP-1 response did not significantly improve model fit compared with the intercept-only model, χ²(1) = 3.13, p = 0.077. In a sensitivity analysis adjusting for fiber category, GLP-1 response again showed a non-significant tendency to improve model fit compared with a model including fiber category alone, χ²(1) = 3.23, p = 0.072.

### 3.2 Resistant starch

With twelve studies, resistant starch (RS) was the most frequently investigated fiber category among the included studies (9–20). Across studies, RS was administered in varying forms (e.g., high-amylose maize starch, resistant wheat starch, RS-enriched bread or rice). Out of the twelve studies, eight had acute study designs with a dose range of 5–48 g per test meal. The dose range in the four non-acute studies was 9.6–45 g per day.

Overall, three of the twelve studies reported a significant effect of RS on at least one satiety-related parameter (9, 17, 19). Only one out of twelve studies reported a positive effect on GLP-1 concentrations in blood (9) following acute intake of 27.1g of the fiber. This was one of the smallest study (study population of n=10) amongst the twelve studies and the only study to use raw potato starch to supplement resistant starch. In contrast, one study reported a decrease in circulating GLP-1 concentrations following supplementation with 5-6g RS derived from high-amylose wheat (15).

### 3.3 Fructans

A total of ten studies investigated the effects of fructans on circulating GLP-1 levels and subjective satiety, including inulin, oligofructose and fructooligosacccharides (FOS) (21–30). Out of the ten studies, four had acute study designs with a dose range of 10–24 g per test meal. The dose range in the six non-acute studies was 10–55 g per day.

Across included studies, effects on circulating GLP-1 were inconsistent. Two studies reported significant increases (21, 23), one study reported a decrease (26), and the remaining seven studies showed no effect on circulating GLP-1. Positive findings were observed in studies administering 16 g/day over a two-week intervention period. Effects on subjective satiety were moderate. Four of the ten studies reported increased subjective satiety for at least one of the parameters (21, 24, 25, 27). These positive satiety responses were generally observed in long-term studies using ≥10 g doses. The other studies showed no satiety-related effect.

### 3.4 Dextrins

Eight studies investigated dextrins on circulating GLP-1 levels and subjective satiety, including polydextrose, soluble fiber dextrin, resistant maltodextrin, and α−cyclodextrin (31–38). Out of the eight studies, seven had acute study designs with a dose range of 5–50 g per test meal. The dose used in the non-acute study was 6.2 g/bar/day.

Dextrins were among the fiber types most associated with increased circulating GLP-1 responses. Four studies demonstrated a significant increase in circulating GLP-1 concentrations (32–35). Moreover, effects on satiety were robust; five out of eight studies reported increased subjective satiety or reduced hunger following dextrin intake (31–33, 35, 38).

### 3.5 β-glucans

A total of nine publications investigated β-glucans (17, 39–46). Out of the nine studies, six had an acute study design with a dose range of 3-20 g per test meal. The dose range of the non-acute studies was 5–9 g/day.

Three of the nine studies reported a significant increase in circulating GLP-1 levels (39, 44, 46), whereas one reported a decrease (42) and the remaining studies showed no effect. In contrast, effects on satiety were more consistent: six out of nine studies reported increased subjective satiety (17, 40–43, 45). This represented the highest percentage of studies supporting a positive effect on satiety among all fiber types evaluated in this review.

### 3.6 Mannans

A total of three publications investigated mannans, including guar gum, and konjac glucomannan (47–49). However, the study by Adam and Westerterp-Plantenga (47) reported two separate acute crossover trials; one conducted in healthy adults and one in individuals with overweight or obesity, resulting in a total of four studies. All four studies had an acute study design and used a dose range of 2.5–5g or 1% w/w mannans.

Three out of four studies reported significant increases in circulating GLP-1 following consumption of galactomannan- or glucomannan-based fibers. Both trials by Adam and Westerterp-Plantenga observed increased GLP-1 responses, although the effect in healthy participants was sex-specific and limited to women. Similarly, Shang et al. reported a direct postprandial increase in GLP-1 following konjac glucomannan intake in healthy adults. In contrast, evidence for effects on subjective satiety was inconsistent, with only two out of the four studies reporting an increased subjective satiety after acute fiber consumption (47, 48).

### 3.7 Arabinoxylan rich

Four studies evaluated arabinoxylan-rich fibers, including psyllium, corn fiber, and rye fiber (10, 50–52). All four studies had an acute study design and used a dose range of 1.7–50 g of arabinoxylan-rich fibers.

Only one study (52) demonstrated an increase in circulating GLP-1 after acute consumption of whole-grain rye kernel bread (containing 50g arabinoxylan-rich starch). Satiety outcomes were mixed: two studies (51, 52) reported increased subjective satiety, while others reported no effect.

### 3.8 Xylans

Three studies examining effects from xylan-containing fibers by using wheat bran extracts that are rich in arabinoxylan-oligosaccharides (AXOS) as intervention products (11, 53, 54). All three studies contained an acute study design and applied a dose range of 3.5–18.4 g of xylans. Two studies reported increases in circulating GLP-1 (53, 54), while the other study found no effect (11). However, none of the studies found a significant effect on subjective satiety.

### 3.9 Pectin

One acute crossover trial investigated a pectin-type fiber (55). In this study, capsules containing 5.6g low-methoxyl pectin were administered. The results showed a significant decrease in GLP-1 concentrations, but a concomitant significant increase in satiety, compared to the blank control group.

### 3.10 Marine polysaccharide

One study examined alginate consumption (2.43g/day) over a three-day period (56). No significant effects were observed on either circulating GLP-1 concentrations or subjective satiety.

## 4 Discussion

The aim of this study was to provide a scoping overview of randomized controlled human trials (RCTs) investigating the effects of dietary fiber supplementation or fiber-enriched foods on endogenous GLP-1 secretion and satiety feeling. Forty−nine publications comprising 52 studies (total n = 1,085 participants; median sample size per study = 19) were included. Most studies (71%) were acute interventions (a single dose), conducted in the USA, United Kingdom or The Netherlands during the period 2016-2020. Across fiber categories, studies reporting increased GLP-1 showed a non-significant tendency to also report increased satiety (OR = 2.95, 95% CI: 0.87–9.98), but GLP-1 response did not significantly improve model fit compared with the intercept-only model (χ²(1) = 3.13, p = 0.077). This inference was similar after adjustment for fiber category, suggesting that the observed tendency was not solely attributable to broad differences between fiber types. Within this framework, dextrins ranked among the higher−performing fiber categories for both outcomes. Other fibers, such as β−glucan and mannans, demonstrated more consistent effects on satiety or GLP−1, respectively, but less frequently on both outcomes simultaneously. Together, these findings suggest that dextrins may warrant particular attention in future research, while acknowledging that the current evidence base remains limited by small sample sizes, short intervention durations, and substantial heterogeneity across studies.

Among the fiber types evaluated, dextrins produced one of the highest numbers of positive findings for both GLP-1 secretion and satiety, with beneficial effects reported in 4 studies for GLP-1 secretion and 5 studies for satiety, out of 8 studies in total. The fibers in this category, such as polydextrose and resistant maltodextrin, are generally more soluble and fermentable (8). The bacterial fermentation of these fibers produces SCFAs which can enter the bloodstream via the portal vein and stimulate the release of endogenous GLP-1 through activation of FFAR2 (21, 57). In addition, SCFAs may slow down gastrointestinal transit, and influence satiety by altering glucose and lipid metabolism (57). The relatively consistent increased GLP-1 responses observed in dextrin interventions may therefore reflect their fermentability and SCFA production. These combined characteristics may explain why dextrins were among the few fibers in this review that affected GLP−1 and subjective satiety in parallel. Consequently, dextrins represent a promising candidate for future long−term trials evaluating whether acute hormonal and satiety effects translate into clinical or metabolic outcomes.

Mannans, including guar gum and glucomannan, demonstrated relatively consistent increases in circulating GLP-1 concentrations (3 out of 4 studies were positive). Their physiological actions extend beyond fermentability: mannans form highly viscous gels in the gastrointestinal tract, which slow gastric emptying and delay nutrient absorption (58, 59). The resulting prolongation of nutrient delivery to the small intestine enhances stimulation of enteroendocrine cells, including L−cells, and may contribute to the observed GLP−1 increases (60). This viscosity−driven mechanism, combined with secondary fermentation processes, provides a plausible explanation for the more uniform GLP−1 responses within this fiber category, despite the more modest and variable effects on subjective satiety.

In contrast, β-glucans demonstrated the most consistent effects on subjective satiety (6 out of 9 studies were positive), despite relatively inconsistent GLP-1 responses (3 out of 9 studies were positive). β-glucans originated from oat and barley sources are highly soluble and form viscous gels in the gastrointestinal tract (61, 62). This slows gastric emptying, prolongs nutrient transit and absorption in the stomach and small intestine (39). These viscosity-mediated effects are well-established drivers of fullness and appetite suppression, providing a plausible explanation for the strong satiety responses observed across the included studies. Taken together, these findings suggest that β-glucans effectively enhance subjective satiety even in the absence of substantial incretin responses, highlighting a mechanism of action that is complementary to, but not necessarily dependent on, GLP-1 stimulation.

The duration of the included studies was notably short, with 71% being acute interventions. This is striking given that most trials therefore do not capture the adaptive responses of the gut microbiota to dietary fiber intake. Studies investigating microbiota dynamics have shown that interventions lasting between four days and three weeks often fail to produce measurable changes in microbial diversity or functional capacity (63). *In vivo* studies suggest that detectable shifts may emerge after approximately four weeks, with more robust and significant changes observed after eight weeks of continuous intervention (64). These findings underscore that the predominance of acute and short term fiber trials may underestimate microbiota mediated effects on GLP-1 secretion, satiety, and metabolic regulation, which likely depend on gradual microbial adaptation. As a result, acute trials reflect SCFA production by the microbiota present at that moment, but may underestimate metabolic effects that emerge only after sustained fiber intake alters microbial composition and function, thereby changing metabolite profiles over time. The predominance of acute studies in the existing literature limits the ability to draw conclusions about longer−term physiological effects, including those related to GLP−1 secretion, appetite regulation, and metabolic homeostasis. At the same time, longer-term studies introduce a different set of challenges. Under free−living conditions, participants’ daily routines vary substantially: dietary intake, meal timing, physical activity, stress, sleep, and general lifestyle behaviors fluctuate both between and within individuals. Such variability can dilute or obscure fiber−specific effects, particularly when studying subjective outcomes like satiety or hormonal endpoints that are sensitive to contextual factors. These real−world influences make long-term studies more reflective of practical use but also less controlled than the tightly standardized acute laboratory (65) settings that dominate the current evidence base. Taken together, the predominance of short-term trials limits conclusions about the sustained physiological effects of fermentable fibers, while the scarcity of well-controlled long-term interventions constrains our ability to determine how these fibers would perform under realistic, everyday conditions. Longitudinal, carefully designed studies are therefore needed to capture both microbiota-mediated adaptation and real-world behavioral variability.

Studies investigating multiple fibers were excluded from the current review to maintain structural clarity and allow meaningful comparisons across distinct fiber categories. However, synergistic effects between fibers remain an important area for future investigation. If stimulation of propionate production is a driver of endogenous GLP-1 production, fiber mixtures could be tailored to the metabolic capabilities of propionate producing microbial taxa, rather than relying on single substrates alone. Mechanistic screening approaches using *in vitro* fermentation models may provide valuable insight into how individual fibers and defined fiber mixtures are metabolized by the gut microbiota prior to translation into human trials. A recent study of Cantu-Jungles et al. demonstrated that microbial fermentation responses differ substantially between single fibers and fiber mixtures, resulting in distinct metabolic outputs (66). Such approaches support the concept that fiber combinations can be designed to target specific microbial pathways, including those involved in propionate production, thereby informing the selection of fiber formulations for subsequent *in vivo* studies. The relevance of these combined effects is further emphasized by real−world dietary patterns, where individuals consume whole foods rather than isolated fiber preparations, and these foods naturally contain complex mixtures of soluble and insoluble fibers with diverse physicochemical properties. Because fibers differ in fermentability, viscosity, solubility, and their capacity to modulate nutrient flow and microbial activity, combining fibers with complementary characteristics may produce physiological effects that exceed those of single−fiber interventions. Such combinations may enhance microbial fermentation, shift SCFA profiles toward more propionogenic or butyrogenic pathways, or prolong stimulation of gut−derived peptides including GLP−1. Future research should therefore examine whether multi−fiber formulations or whole−food matrices elicit stronger or more sustained effects on GLP−1 secretion, satiety, and related metabolic outcomes than isolated fibers alone.

A strength of this study was that all included studies had to report both GLP−1 and satiety outcomes, which allows us to compare hormonal and subjective responses side by side, an angle that has been missing in earlier work. Another strength was classification of fibers based on structural characteristics which allowed comparison across diverse fiber types within a consistent framework. This review also has limitations. First, the included studies varied widely in design, participant characteristics, fiber doses, and product formulations, which makes direct comparison not easy. The substantial heterogeneity among studies impeded the ability to rigorously evaluate dose-response relationships, as variations in dose were frequently confounded by differences in intervention duration, study population, and fiber characteristics. As a result, it was not possible to isolate dose-dependent effects across studies. Although we adjusted for fiber category as a fixed effect in a sensitivity analysis, this adjustment did not materially alter the GLP-1–satiety inference and the association remained non-significant. The sparse distribution of observations across fiber categories also limited the complexity of the statistical models that could be fitted. In particular, the data did not support more complex random-effects models accounting for fiber category or study-level clustering. Second, most studies had small sample sizes, with a median of 19 participants per study, increasing the chance of inconsistent results. Third, the evidence is dominated by short-term, tightly controlled acute trials, which do not reflect longer-term effects that depend on gradual microbiota adaptation. The few longer studies that were available were conducted under free−living conditions, where day−to−day variation in diet and behavior can blur fiber−specific effects. Fourth, no formal assessment of study quality or risk of bias was performed, which should be considered when interpreting the findings. Fifth, the fiber classification used in this review was adapted from Wanders et al. and was chosen to balance chemical specificity with the need for meaningful synthesis across studies. We acknowledge that individual fibers within a category may differ in physicochemical properties such as molecular weight, branching structure, viscosity, and fermentability, which may influence GLP-1 secretion and satiety responses. However, these characteristics were inconsistently reported, preventing a more granular classification. Finally, by excluding studies that combined multiple fibers, the review does not fully capture how fibers are consumed in real diets.

Future research should prioritize longer-term randomized controlled trials that allow time for meaningful microbiota adaptation and more stable patterns of endogenous GLP-1 production to emerge. Such studies would help determine whether the short−term hormonal and satiety effects observed in acute trials persist, strengthen, or change once the gut ecosystem has adjusted to sustained fiber intake. In addition, trials that test combinations of fibers with complementary properties, such as pairing fermentable fibers with viscous ones, may provide insight into whether blended formulations produce stronger or more durable effects than single fibers alone. Designing studies that compare isolated fibers with whole−food matrices could also help clarify how real−world eating patterns influence microbial fermentation, GLP−1 secretion, and appetite regulation. Such approaches would help determine which types or combinations of fibers are most effective in sustaining appetite regulation and metabolic benefits during GLP−1RA tapering and the transition to long−term lifestyle-based maintenance. Furthermore, only few studies measured habitual or baseline dietary fiber intake, limiting insight into whether observed effects occurred against an already adequate or generally low fiber consumption. Given that habitual dietary fiber intake in Western populations remains below recommended levels (67), most intervention studies may evaluate fiber effects against an already low baseline intake. This suggests that short-term trials may misestimate the effects that could emerge under conditions of sustained fiber intake. In addition, the effects of fibers on GLP-1 secretion may not solely be determined by the physicochemical properties of the fiber itself, but also by the composition and metabolic capacity of the individual’s gut microbiome. The presence of bacterial taxa capable of fermenting specific fibers into propionate and other SCFAs may modulate the GLP-1 response to fiber intake (68). Therefore, in future research it would be of interest to measure baseline microbiome composition as well to account for this potential effect. Lastly, highly fermentable fibers such as dextrins and fructans may induce gastrointestinal symptoms (e.g., bloating, flatulence, abdominal discomfort), particularly when rapidly fermented in the proximal colon. These effects are more pronounced in individuals with functional bowel disorders or visceral hypersensitivity. The severity of symptoms is likely dose-dependent and may be mitigated by gradual dose escalation and microbiota adaptation. However, tolerability may still be limited in certain populations. Future studies should therefore systematically assess gastrointestinal side effects and adherence alongside metabolic outcomes.

In conclusion, this scoping review shows that dietary fibers differ markedly in their capacity to influence endogenous GLP−1 secretion and subjective satiety. Among the fiber categories examined, dextrins consistently produced parallel effects on both outcomes, based on the currently available, yet limited, body of evidence. Mannans and β−glucans showed more selective effects on GLP−1 or satiety, respectively. RS was the most frequently investigated fiber, but showed limited and inconsistent effects on subjective satiety and circulating GLP-1 levels. However, the current evidence base remains limited by small sample sizes, heterogeneous study designs, and a predominance of short-term, acute trials that do not allow for microbiota adaptation or assessment of sustained effects. To move the field forward, longer-term, well−controlled studies are needed to determine whether the short−term GLP−1 and satiety responses observed here translate into sustained appetite regulation and metabolic support. Such research will be particularly relevant for individuals tapering GLP−1RA therapy, for whom dietary strategies that support endogenous GLP−1 production and minimize weight regain are urgently needed.

## Data Availability

The original contributions presented in the study are included in the article/**Supplementary Material**, further inquiries can be directed to the corresponding author/s.

## Supplementary material

Supplementary Table 1 Overview of the major dietary fiber categories included in this review (based on 8), including their structural characteristics, physicochemical properties, fermentability, and proposed physiological effects related to satiety and colonic fermentation.

Supplementary Table 2 An overview of all included studies in this scoping review, including the extracted data from each study.
