# Supplementary material for: Dietary fibers to boost endogenous GLP-1 secretion and satiety: a scoping review
Source: Front Endocrinol (Lausanne). 2026 Jul 15;17:1880500. doi: 10.3389/fendo.2026.1880500 (PMC13414124; doi:10.3389/fendo.2026.1880500)
Supplement: Supplementary Table 1 — Overview of the major dietary fiber categories included in this review (based on 8), including their structural characteristics, physicochemical properties, fermentability, and proposed physiological effects related to satiety and colonic fermentation. [file Table1.docx]

| **Fiber category** | **Main structural characteristics** | **Solubility / viscosity** | **Fermentability** |
| --- | --- | --- | --- |
| Resistant starch | α-1,4-linked glucose polymers | Poorly soluble | Fermentable |
| Dextrins | α-linked glucose polymers | Soluble | Fermentable |
| β-glucans | β-linked glucose polymers | Moderately soluble | Variable |
| Mannans | Mannose-based polymers | Soluble, highly viscous | Moderately fermentable |
| Fructans | β-linked fructose polymers | Soluble | Highly fermentable |
| Xylans | Xylose-based polymers | Variable solubility | Variably fermentable |
| Arabinoxylan-rich fibers | Cereal-derived xylans | Less soluble | Fermentable |
| Pectins | Galacturonic acid polymers | Soluble, viscous | Highly fermentable |
| Marine polysaccharides | e.g. alginates | Soluble, highly viscous | Variable |

**Supplementary table 1:** Overview of the major dietary fiber categories included in this review (based on Wanders et al., 2011), including their structural characteristics, physicochemical properties, fermentability, and proposed physiological effects related to satiety and colonic fermentation.
